# Effect of enteral and/or parenteral glutamine supplementation on mortality and morbidity in the critically ill

**DOI:** 10.1186/cc13614

**Published:** 2014-03-17

**Authors:** H Sungurtekin, I Ozturk, B Beder, S Serin

**Affiliations:** 1 Pamukkale University Medical Faculty, Denizli, Turkey

## Introduction

In this study we aimed to compare the effectiveness of enteral, parenteral and combined enteral-parenteral glutamine supplementations in the nutrition of critical care patients.

## Methods

This is a single-center, randomized controlled clinical trial. During the 5-day study period, all patients received standard enteral nutrition product and were divided into three groups, including parenteral glutamine (Group I), enteral glutamine (Group II) and enteral + parenteral glutamine (Group III) supplementations. Blood biochemistry, rates of infections, length of stay in the ICU and duration of mechanical ventilation were evaluated.

## Results

Sixty patients were included in this study. There was no statistically significant difference for biochemical values between the different feeding groups. Frequency of infections ranged as Group II >Group III >Group I and mortality as Group II = Group III >Group I. Length of stay in the ICU and duration of mechanical ventilation were significantly longer in Group II than the others.

## Conclusion

Although mortality was not significantly different between groups, parenteral glutamine administration causes less stay of ICU and mechanical ventilation. This needs a more powerful randomized controlled study [1].
